# The advanced development of molecular targeted therapy for hepatocellular carcinoma

**DOI:** 10.20892/j.issn.2095-3941.2021.0661

**Published:** 2022-06-15

**Authors:** Tao Yan, Lingxiang Yu, Ning Zhang, Caiyun Peng, Guodong Su, Yi Jing, Linzhi Zhang, Tong Wu, Jiamin Cheng, Qian Guo, Xiaoliang Shi, Yinying Lu

**Affiliations:** 1 Comprehensive Liver Cancer Center, the 5th Medical Center of Chinese PLA General Hospital, Beijing 100039, China; 2 The Second School of Clinical Medicine, Southern Medical University, Guangzhou 510515, China; 3 The Second Department of Hepatobiliary Surgery, Senior Department of Hepatology, the 5th Medical Center of Chinese PLA General Hospital, Beijing 100039, China; 4 Shanghai OrigiMed Co., Ltd., Shanghai 201112, China; 5 National Clinical Medical Research Center for Infectious Diseases, the 5th Medical Center of Chinese PLA General Hospital, Beijing 100039, China

**Keywords:** Hepatocellular carcinoma, precision medicine, liquid biopsy, targeted therapy, immunotherapy

## Abstract

Hepatocellular carcinoma (HCC), one of the most common malignant tumors in China, severely threatens the life and health of patients. In recent years, precision medicine, clinical diagnoses, treatments, and innovative research have led to important breakthroughs in HCC care. The discovery of new biomarkers and the promotion of liquid biopsy technologies have greatly facilitated the early diagnosis and treatment of HCC. Progress in targeted therapy and immunotherapy has provided more choices for precise HCC treatment. Multiomics technologies, such as genomics, transcriptomics, and metabolomics, have enabled deeper understanding of the occurrence and development mechanisms, heterogeneity, and genetic mutation characteristics of HCC. The continued promotion and accurate typing of HCC, accurate guidance of treatment, and accurate prognostication have provided more treatment opportunities and prolonged survival timelines for patients with HCC. Innovative HCC research providing an in-depth understanding of the biological characteristics of HCC will be translated into accurate clinical practices for the diagnosis and treatment of HCC.

## Introduction

Liver cancer is the fourth leading cause of cancer-associated death worldwide, accounting for more than 840,000 new cases and 780,000 deaths each year^1,2^. According to the World Health Organization, the number of deaths from liver cancer will exceed 1 million by 2030, thus posing a major threat to human health^3^. The 2 most frequent forms of liver cancer are hepatocellular carcinoma (HCC) and cholangiocarcinoma, which represent 85%–90% and 6%–15% of all primary liver cancers, respectively^4,5^.

HCC’s late symptom manifestation leads to delayed diagnosis, thus limiting curative surgical treatments. Consequently, HCC is one of the deadliest cancers^6^. HCC generally develops in the context of liver cirrhosis due to any cause, and the most common underlying etiologies are hepatitis B virus (HBV) or hepatitis C virus (HCV) infection, and alcoholic or nonalcoholic fatty liver disease^7,8^. Worldwide, regional differences exist regarding the average age of onset of liver cancer^9^. In Asian and African countries, the age of onset is generally 30–60 years^10^. A Chinese study in 14,891 patients with HCC from 2016 to 2018 has found that the incidence rate of HCC significantly increases at the age of 40, particularly in men^11^. Screening for risk, including by age and for hepatitis virus infection, is helpful for improving early diagnosis. Over the past few decades, findings regarding epidemiology, risk factors, and molecular and genetic characteristics have contributed to strategies for the prevention, surveillance, early diagnosis, and treatment of liver cancer^12^. Although they require diagnosis during early stages, liver resection, ablation, and liver transplantation have potential efficacy in the treatment of liver cancer^13^.

Precision medicine, also known as stratified medicine or personalized medicine, aims to tailor interventions to individual patients to maximize benefits and minimize harm^14^. With major advances in cancer genomics through next-generation sequencing (NGS) technologies, precision medicine based on genomic and molecular profiling is now used as part of routine clinical testing to guide and select the most appropriate treatments for individual cancer patients^15^. In cancer research, personalized medicine based on genomics and pharmacogenomics is rapidly expanding. With a multiomics approach, patients are no longer treated only on the basis of tumor histology; instead, actionable targets specific to an individual’s tumor biology are used^16^.

Two streams of precision medicine development are of interest to the medical community. The first involves the integration of electronic medical records that capture longitudinal data and indicate clinical phenotypes for decision-making. The second involves advances in genomic medicine and pharmacogenomics research that provide an expanding arsenal of genetic predictors for disease and health outcomes^14^. NGS highlights tumor molecular heterogeneity and challenges the one-size-fits-all treatment paradigm, yet also offers insights regarding potential tumor vulnerabilities that can be exploited^16^. Here, to consider new ideas regarding precision treatments and research for liver cancer, we review advanced developments in precision medicine for HCC, including early diagnosis and treatment, biomarkers for diagnosis and prognosis, advanced targeted therapy and immunotherapy, and expectations of precision medicine for HCC.

## Early diagnosis and treatment of HCC

Early diagnosis of liver cancer includes a risk assessment of clinical indicators such as the age-male-albumin-bilirubin-platelet system^17^, alpha-fetoprotein (AFP), and des-gamma carboxyprothrombin^18,19^. Imaging examinations such as abdominal ultrasound, computed tomography, and magnetic resonance imaging are also used^20^. However, because the sensitivity and specificity of ultrasound and AFP are 63% and 84%, respectively, these values are not suitable for the early detection of HCC^21^.

With the development of molecular biology technology, molecular testing has been used as a supplement in individualized diagnosis. The lack of available tissue for analysis of molecular mutations and the biological processes associated with tumorigenesis is the main factor influencing the development of effective and accurate treatment methods for HCC. Circulating free DNAs (cfDNAs) are free DNA fragments released into the blood by cells. cfDNA in plasma has gained global interest as a material for cancer diagnosis and is often called a “liquid biopsy”^22^. Many studies have demonstrated that tumor related variations can be observed within the cfDNA of cancer patients. However, a lack of effective molecular markers prevents current guidelines from recommending routine diagnostic biopsies; consequently, simple imaging examinations are currently used for the diagnosis and treatment requirements for HCC^23^.

HCC is highly heterogeneous both intra- and inter-tumorally^24,25^. Precise classification based on molecular characteristics is required in early stages for precise HCC medical treatment. Multiomics techniques such as transcriptomics, genomics, proteomics, and metabonomics have been applied for the molecular typing of HCC^26,27^ (**Table 1**). Transcriptome based HCC typing typically considers the gene expression characteristics of different subtypes. For example, Lee et al.^28^ have classified HCC with the expression of cell proliferation and antiapoptosis related genes as subtype A, and HCC with upregulated expression of ubiquitination and histone modification related genes as subtype B. Boyault et al.^29^ have classified HCC into 6 subtypes (G1–G6) according to the overexpression of fetal liver genes, *PIK3CA* mutation, *TP53* mutation, AKT pathway activity, overexpression of cell cycle related genes, WNT pathway activity, and β-catenin activity. Hoshida et al.^30^ have classified HCC into 3 subtypes (S1–S3) according to the activity of the TGF-β and WNT pathways, activity of the MYC and AKT pathways, overexpression of stemness markers (including AFP and epithelial cell adhesion molecule), and overexpression of liver function related genes. According to the gene expression profiles of metabolic genes, Yang et al.^31^ have established a new HCC classification of C1–C3. The C1 subclass has high metabolic activity, low AFP expression, and good prognosis; the C2 subclass has low metabolic activity and high expression of immune checkpoint genes; and the C3 subclass has moderate metabolic activity, high AFP expression, and poor prognosis. Gene expression profiling for metabolic genes provides a basis for new HCC classifications, thus increasing understanding of the genetic diversity of human HCC.

**Table 1:** Molecular classifications for HCC

Omics	Patient No.	HCC subtype	Meaning	Reference
Genomic	300	C1–C6	Clinical implications and association with prognosis	Fujimoto et al., 2016^33^
Genomic	243	MSig 1–MSig 6	Design of clinical trials for targeted therapy	Schulze et al., 2015^32^
Transcriptome	91	A, B	Prediction of HCC clinical outcome at the time of diagnosis	Lee et al., 2004^28^
Transcriptome	123	G1–G6	Identification of patients who may benefit from targeted therapies	Boyault et al., 2007^29^
Transcriptome	603	S1–S3	Guidance for the design of future clinical trials aimed at targeting agents to distinct patient populations	Hoshida et al., 2009^30^
Transcriptome	231	C1–C3	Prediction of the prognosis of patients with HCC and prospective therapies	Yang et al., 2020^31^
Proteome	159	S-Mb, S-Pf, S-Me	Provision of unique therapeutic opportunities	Gao et al., 2019^34^
Proteome	110	S-I, S-II, S-III	Provision of insight into tumor biology and opportunities for personalized targeted therapies	Jiang et al., 2019^35^

The identification of tumor genomic alterations may potentially improve the survival of patients by guiding targeted treatments and the classification of HCC subtypes. On the basis of mutational characteristics, HCC can also be classified into 6 subgroups, MSig 1–6^32^. Fujimoto et al.^33^ have classified HCC into 6 subtypes according to the genomic profiling of 300 HCCs. The mutational characteristics of these subtypes included: (1) patients with *ARID2* and *PBRM1* mutations, (2) patients with *LRP1B*, *ARID1A*, *PTPRD*, *RB1*, and *APOB* mutations, (3) patients with *MACDROD2* mutations, (4) patients with *CTNNB1* mutations, (5) patients with *CDKN2A* mutations, and (6) patients with *TP53* mutations^33^.

Proteome based typing is usually performed according to different signal characteristics and metabolic pathways. Gao et al.^34^ have divided HCC into subtypes of highly expressed proteins associated with liver function metabolism; up-regulated differentiation related proteins; and down-regulated immunity, inflammation, and stromal proteins. Jiang et al.^35^ first divided HCC into S-I, S-II, and S-III subtypes by using quantitative proteomics data. Despite the achievements of these studies, challenges remain in translating molecular subtypes to clinical practice^36,37^. Although the S2 subtype responds to a small molecular bromodomain and extra-terminal bromodomain inhibitor^38,39^, different subtypes of HCC may vary in their responses to molecular target agents. Therefore, accurate molecular typing is helpful for precision medicine and in the future may become an effective tool for guiding precision medicine. More research is needed regarding how to apply precision medicine to molecular typing and early diagnosis for HCC.

Circulating tumor cells (CTCs) are tumor cells in the peripheral blood that spread during early stages of disease^40^. CTC detection technology measures the presence of CTCs in the peripheral blood by capturing and detecting CTCs, which are then used to monitor tumor dynamics, evaluate treatment outcomes, and determine individual treatments in real time. Clinical studies have confirmed that this technology can be used for early diagnosis, and the prediction of postoperative metastasis and the recurrence of liver cancer^41^. Guo et al.^41^ have generated a multi-marker CTC detection panel showing greater potential than AFP for diagnosing early-stage HCC. Their results have suggested that the CTC panel is a novel biomarker detection tool for the early diagnosis of HCC and complementary diagnostic protocols. In terms of differential diagnostic capability, CTC outperforms AFP as a biomarker, yielding a higher area under the curve, higher sensitivity, and higher specificity for HCC^41^. Improved CTC detection systems can be used to analyze the genomic information for a single CTC through single cell sequencing and can also assist in the differential diagnosis of malignant tumors^42^. Therefore, the early detection of CTC in the blood plays an important role in prognostication, and the evaluation of curative effects and individualized treatments for patients with HCC.

Surgical treatment, the first choice for the early treatment of liver cancer: includes (1) surgical resection (local resection can be performed for cancerous liver lobes), (2) liver transplantation (if a patient has cirrhosis and a tumor, and if the tumor size is suitable for transplantation), and (3) minimally invasive treatment (if a patient does not receive a liver transplant, and surgery has contraindications). The most commonly used minimally invasive treatment for early HCC is ablation therapy, including radiofrequency ablation (RFA) or microwave ablation, and hepatic artery intervention and chemotherapy^43^. For patients with HCC and early-stage HCC without surgical contraindications, liver resection or liver transplantation is the preferred therapy^15,44^. Limitations of RFA include the heat-sink effect, the RFA cytotoxic capacity, and its restriction, according to tumor location^45^. Such treatments are prescribed for patients with early stage disease^43^ Surgical treatment offers a potentially curative option for patients with HCC. However, patient outcomes vary because of differing tumor characteristics. Because the exact biology of HCC remains poorly understood, predicting outcomes of surgical resection remains very difficult^46^.

## Biomarkers for HCC diagnosis and prognostication

Biomarkers are biochemical indicators of changes in the structure or function of systems, organs, tissues, cells, and subcellular organelles^47^. For HCC, biomarkers including classical clinical markers, molecular biomarkers, and emergent immune biomarkers, can be applied for early detection for HCC, and prediction of HCC prognosis and treatment response.

Clinical markers for HCC include clinical indicators of extrahepatic extension or large vessel invasion, inflammatory response, and muscle condition. The European Association for liver research has shown that extrahepatic extension or large vessel invasion is associated with poor prognosis^48^. The SHARP trial has also indicated that vascular invasion is an independent predictor of poor prognosis of HCC^49^. Inflammation related indexes in HCC, including the neutrophil lymphocyte ratio, the systemic immune inflammation index, and the Glasgow prognosis score, can be used as biomarkers for predicting prognosis. Studies have shown an association between poor prognosis and a high neutrophil-to-lymphocyte ratio, thus suggesting that this ratio may serve as a negative prognostic indicator for advanced patients with HCC^50,51^.

Sarcopenia is associated with systemic inflammation. Many studies have reported that sarcopenia is associated with poor HCC prognosis and serves as a negative prognostic marker^52,53^. Additionally, HBV/HCV infection may also serve as a clinical biomarker. For example, Bruix et al.^54^ have found that sorafenib treatment may potentially lead to poorer overall survival (OS) in patients with HCC without HCV infection. However, clinical markers are based on existing symptoms and provide minimal treatment guidance.

With the advancement of molecular biology research, biomarkers based on molecular characteristics have been widely explored and have promoted precision medical treatments for HCC. AFP is closely associated with the occurrence and development of HCC, and AFP levels are used as a serum marker for diagnosis and treatment efficacy monitoring of primary HCC^55^. AFP based diagnostic methods remain far from meeting clinical needs. The identification of serum AFP cannot distinguish between increased AFP caused by other factors such as liver cirrhosis, or chronic hepatitis caused by HBV or HCV infection. However, a high level of AFP is associated with poor OS for HCC^49,56^. Although nearly 30%–40% of patients with HCC are negative for AFP, this biomarker is widely used for HCC prognostication^21,57^.

Emerging biomarkers based on molecular characteristics have been found to effectively aid in the diagnosis of tumor heterogeneity and HCC treatment decision-making. Genomic landscape analysis enables the possibility of developing molecular biomarkers. Research on the genomic landscape of HCC has led to the identification of several significantly mutated genes, including the tumor suppressor genes *TP53*, *AXIN1*, and *RB1*; the WNT pathway oncogene *CTNNB1*; and the chromatin remodeling genes *ARID1A*, *ARID2*, and *BAP1*^32,58,59^. *TP53* encodes the p53 protein and plays important roles in cell cycle regulation, cell migration, the DNA damage response, and angiogenesis. Researchers have also reported that the abnormal accumulation of p53 protein can lead to production of anti-p53 antibodies in serum, tissues, and cells^60,61^; moreover, the positivity rate for anti-p53 antibody is 93.3% in HCC patients with p53 mutations^62,63^. A meta-analysis has indicated that the low sensitivity of antibodies to p53 limits their clinical application^64^. Thus, although, p53 is not currently helpful for the early diagnosis of liver cancer, in the future, its high specificity for HCC may play an important role in exploration of HCC precision medicine.

*CTNNB1* encodes a beta catenin protein that plays an important role in cell-cell adhesion and gene transcription^65^. CTNNB1 ctDNA is a biomarker for sporadic hepatoblastoma treatment response that has great clinical value^66^. CTNNB1 mutation may lead to activation of the WNT signaling pathway^67^. Studies have shown that patients with WNT pathway mutations, including CTNNB1 mutations, have a poor response to targeted therapy and immunotherapy^68^. These results also support the potential value of *CTNNB1* as a therapeutic biomarker.

Mutations in the promoter region of the telomerase reverse transcriptase (TERT) gene are often found during early stages of HCC and are considered the key driver of HCC^69^. The *TERT* C228T promoter mutation is the most common point mutation found in patients with HCC. A *TERT* C228T mutation in ctDNA is considered a promising prognostic biomarker for HCC^70,71^. However, regional specificity is known to exist for the *TERT* promoter mutation. The mutation frequency for the *TERT* promoter is higher in patients from Western countries than those from East Asia^70^. A negative correlation between *TERT* promoter mutation and HBV infection is also known to exist^72^ and may explain differences in *TERT* mutation frequencies due to population specificity.

More potential biomarkers have also been identified through association analyses between genomic alteration and the prognosis or response to drug treatment. For example, the FGF19 level can be used as a targeted biomarker for predicting treatment response to lenvatinib in patients with unresectable liver cancer^73^. Myojin et al.^74^ have identified a novel potential biomarker, ST6GAL, for identifying lenvatinib-susceptible FGF19-driven HCC. However, very few reported molecular biomarkers have been externally validated.

Immunological biomarkers are also emerging for HCC. Ye et al.^75^ have found that agrocybe aegerita galectin induces the activation and migration of lymphocytes to the liver, and that the combination of agrocybe aegerita galectin and anti-PD-1 may be a promising strategy for HCC treatment. Programmed-death ligand 1 (PD-L1) is the ligand of immune checkpoint receptor programmed-death 1 (PD-1). The expression of PD-L1 is associated with poor HCC prognosis^76^. However, tumor cells and associated stromal cells, as well as T effector cells, also express this checkpoint protein^77^. In a clinical trial of CheckMate-040 and KEYNOTE-224, the expression of PD-L1 in tumor cells and macrophages in HCC has not been found to have strong predictive value for therapeutic response^78,79^. A recent study has also confirmed that the response of anti-PD-1 is not consistent with PD-L1 expression in tumor tissues^80^. However, the spatial heterogeneity of PD-L1 expression and the different cut-off values used for evaluating positive staining have led to differences in PD-L1 expression in tumors assessed by immunohistochemistry^81,82^. Further research is required to obtain a unified standard for evaluating PD-L1 expression suitable for HCC.

The tumor mutational burden (TMB) is defined as the number of somatic mutations within a tumor genome^83^. The TMB is considered a promising biomarker for predicting the efficacy of immunotherapy, and is associated with high neoantigen expression, T-cell infiltration, and checkpoint inhibitor response rates in multiple tumor types^84,85^. Several studies have indicated that PD-L1 and TMB are independent predictors of the immune checkpoint blockade response, and PD-L1 expression and TMB have a low correlation across multiple tumor types, including HCC^86–88^. In contrast, Xu et al.^89^ have reported that PD-L1 positive patients exhibit a lower TMB than PD-L1 negative patients, and that PD-L1 positive patients are more likely to have aggressive clinicopathological features than PD-L1 negative patients. A model of 15 immune-associated gene pairs associated with TMB has been developed for prediction of prognosis^90^. Owing to the limitations of PD-L1 and TMB as biomarkers, determining the independent benefit of TMB in predicting responses to anti-PD-1/PD-L1 therapy would be highly clinically useful. The expression of immune-associated genes has also been considered a biomarker for prognosis prediction. Wang et al.^91^ have classified HCC subtypes according to the increased expression of immune-associated genes and corresponding poor prognosis. Du et al.^92^ have described a specific biomarker for the prediction of OS on the basis of the expression of 5 immunity-associated and 2 AFP-associated genes.

In conclusion, biomarkers display a wide range of applications, including associations with early diagnosis, adjuvant therapy, and prognostication. However, the heterogeneity of HCC in different populations prompts the question of whether the same biomarkers are effective for different HCC populations. This unanswered question remains a bottleneck in the application of many new biomarkers and should be confirmed through clinical studies. The development of biomarkers in the future should also be confirmed through clinical studies.

## Advanced progress in targeted therapy for HCC

**Figure 1:**
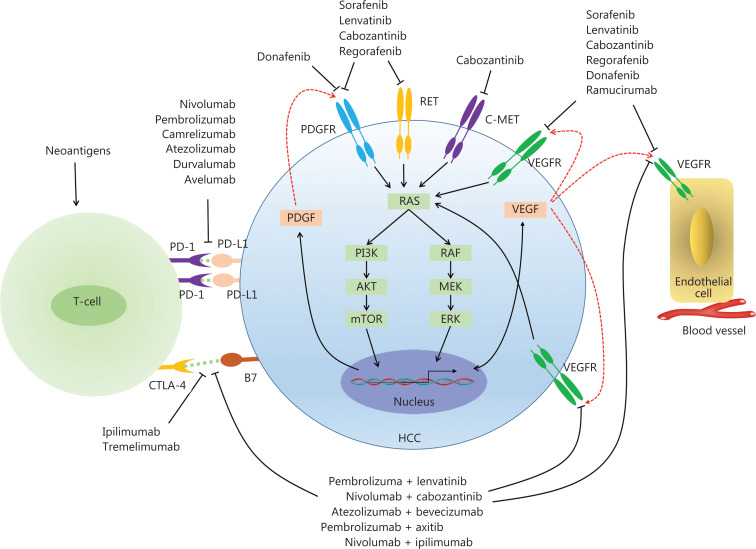
Patterns of targeted therapy, immunotherapy, and combined immunotherapy in HCC.

HCC is highly heterogeneous, showing substantial differences in molecular characteristics among patients^24^. This heterogeneity results in limited systematic treatments for patients with HCC. Targeted therapy has the advantage of clear targeting and low toxicity, and can effectively improve HCC treatment (**Table 2**). A better understanding of HCC molecular biology, resulting from an increased number of druggable targets, such as intracellular signal proteins, angiogenesis factors, peptide growth factors and their receptors, cell cycle regulators, and transcription factors, has been gained through several studies^93–95^. Antiangiogenic agents are an effective type of molecular targeted therapy for HCC. The vascular endothelial growth factor (VEGF)/VEGF receptor (VEGFR) pathway is the main signaling pathway in tumor angiogenesis^96^ (**Figure 1**). VEGF/VEGFR pathway targeted therapy strategies are classified with multi-target kinase inhibitors and monoclonal antibodies.

**Table 2:** Targeted therapy in HCC

Drug	Control	Line	Status	Reference
Sorafenib	Placebo	First line	Approved (US FDA, NMPA)	Llovet et al., 2008^97^; Cheng et al., 2009^98^
Lenvatinib	Sorafenib	First line	Approved (US FDA, NMPA)	Kudo et al., 2018^115^
Sunitinib	Sorafenib	First line	Failed	Cheng et al., 2013^112^
Linifanib	Sorafenib	First line	Failed	Cainap et al., 2015^113^
Dovotinib	Sorafenib	First line	Failed	Cheng et al., 2016^144^
Donafenib	Sorafenib	First line	Approved (NMPA)	Bi et al., 2020^119^
Apatinib	Sorafenib	Second line	Approved (NMPA)	He et al., 2020^134^
Regorafenib	Non-candidate groups	Second line	Approved (US FDA, NMPA)	Kuzuya et al., 2019^123^; Teufel et al., 2019^122^
Cabozantinib	Non-candidate groups	Second line	Approved (US FDA)	Kuzuya et al., 2019^123^; Abou-Alfa et al., 2018^126^
Ramucirumab	Placebo	Second line	Approved (US FDA, NMPA; ongoing)	Zhu et al., 2015^130^; Kudo et al., 2020^132^

Sorafenib, a VEGFR family multi-kinase inhibitor, was the first approved systemic drug for the treatment of advanced HCC. SHARP and Asia Pacific studies have shown that sorafenib benefits patients with liver cancer^97,98^ and has an antitumor effect on recurrent tumors after liver transplantation^99,100^. HCC that initially cannot be resected can be phased down after sorafenib treatment and therapeutic surgery^101^. Studies have also shown that baseline liver function, clinicopathological features, and etiology also affect the prognosis of patients with liver cancer treated with sorafenib^102^. Sorafenib and transarterial chemoembolization (TACE) are both recommended therapies for advanced HCC. However, whether the combination of sorafenib and TACE might benefit patients with HCC remains controversial. The heterogeneity of HCC caused by different populations may potentially affect treatment results. For example, in a phase 3 European trial, no significant difference has been found between TACE alone and the combination of TACE and sorafenib^103^. However, studies from China have shown that a combination of sorafenib and TACE, compared with TACE alone, results in more than 50% higher OS ^104,105^. Recently, the TACTICS trial from Japan has also found that TACE plus sorafenib significantly improves progression free survival (PFS) over that of TACE alone in patients with unresectable HCC^106^. The addition of sorafenib has also been found not to confer a survival benefit in patients with unresectable HCC that has already responded to TACE^107^.

Regarding sorafenib, the clinical outcomes might have been due to problems regarding how to use the drug and how to obtain earlier drug effects. The prediction of outcomes for sorafenib therapy by using biomarkers is an unmet clinical need for patients with advanced HCC. Biomarkers such as *FGF3/FGF4* gene amplification and galectin-1 have been evaluated and validated for guiding treatment ^108^. A new model including serum FGF and HGF has shown good performance for sorafenib in predicting the response and survival of patients with advanced HCC^109^. Additionally, Kim et al.^110^ have explored a triple-marker panel for predicting the response of sorafenib in patients with HCC. However, the panel is not suitable for patients treated with TACE.

Antiangiogenic agents including MEK/ERK pathway inhibitors, mTOR pathway inhibitors, histone deacetylase inhibitors, EGF/EGFR pathway inhibitors, and HGF/c-Met pathway inhibitors have also been used in combination with sorafenib for the treatment of HCC, and have been determined to achieve relatively positive results^111^.

Following the sorafenib trial, many clinical trials for first-line HCC drugs have been performed with sorafenib as a control. From 2007 to 2017, many clinical studies were performed on treatments such as sunitinib, linifanib, and dovotinib; however, none of these treatments show greater efficacy than solafenib in improving HCC survival/prognosis ^112–114^.

Lenvatinib, a multikinase inhibitor of the VEGFR family of protein, fibroblast growth factor receptor, PDGFR-α, and KIT and RET inhibitors, was the second approved first-line targeted drug for advanced HCC. A phase III reverse clinical trial has found that the median OS with lenvatinib treatment was not lower than that with sorafenib (13.6 months *vs.* 12.3 months, respectively)^115^. However, lenvatinib results in significantly longer OS (37.9 months *vs.* 21.3 months) and PFS (16.0 months *vs*. 3.0 months) in patients with intermediate liver cancer whose tumors exceed 7 criteria^116^. Lenvatinib is known to cause thyroid toxicity. Therefore, thyroid abnormalities should be monitored during treatment^117^. In addition, lenvatinib is more cost-effective than sorafenib^118^.

Donafenib is a new multikinase inhibitor. Bi et al.^119^, in a randomized phase II/III clinical trial within the Chinese population, have found a significantly longer OS in the donafenib group than the sorafenib group. Thus, donafenib significantly prolongs the OS in Chinese patients with advanced HCC, and shows good safety and tolerance.

Second-line targeted agents for HCC include regorafenib, cabozantinib, and ramucirumab. Regorafenib is a multi-kinase inhibitor that targets the VEGFR protein family, PDGFR-β, B-RAF, c-KIT, FLT3, and RET. Research studies have shown significant benefits for patients with HCC who tolerate and show disease progression on sorafenib^120,121^. Teufel et al.^122^ have identified biomarkers for expression patterns of plasma proteins and miRNAs associated with longer OS in patients with HCC after regorafenib treatment in sorafenib tolerant patients. However, not all patients with HCC with sorafenib tolerance can receive regorafenib treatment^123,124^. Good liver function reserve and Eastern Cooperative Oncology Group Performance Status performance during sorafenib treatment may contribute to efficacy and better results during follow-up treatments^124,125^.

Cabozantinib is another multikinase inhibitor that is effective in patients with HCC with sorafenib tolerance^126^. Cabozantinib blocks receptors involved in tumorigenesis and angiogenesis *in vitro* and *in vivo*, including the VEGFR protein family; the hepatocyte growth factor receptor (MET); and the AXL and angiopoietin receptors Tie-2, RET, c-KIT, and FLT-3. However, because of its high cost, few studies have examined cabozantinib as a second-line drug^127,128^.

Ramucirumab is a human recombinant IgG1 monoclonal antibody that targets the VEGF2 receptor^129^. Patients receiving ramucirumab did not reach the endpoint in second-line treatments during early clinical trials^130^. However, in the REACG-2 trial, which led to the approval of ramucirumab as a second-line treatment for advanced HCC, the results confirmed that ramucirumab may improve the survival of patients with HCC with an AFP greater than 400 ng/mL^131,132^. Studies have also indicated that patients with high AFP and HCC display substantial activation of VEGF, thus indicating the potential mechanism of action and providing a theoretical basis for VEGF targeted therapy^133^. Regarding additional second-line treatments, a clinical study of apatinib in Chinese patients with advanced HCC has found comparable PFS and OS, and a better objective response rate (ORR) than those with sorafenib^134^. With the goal of obtaining approval for apatinib as a second-line treatment for advanced HCC, data obtained from the study have been submitted to the National Medical Products Administration (NMPA) (China).

The inhibition of VEGF/VEGFR is often accompanied by certain adverse effects^135^. With multikinase VEGFR inhibitors, adverse effects often occur to different degrees, such as hand-foot-skin reactions, diarrhea, and fatigue, which must be managed through dose adjustments or altered administration schemes^136^.

Advances in molecular cell biology have contributed to knowledge of the molecular mechanisms of tumorigenesis and its progression, and have provided opportunities for the development of novel molecular targeting agents. Molecular targeted therapy mainly includes tyrosine kinase inhibitors (TKIs) and/or monoclonal antibodies. The combination of TKIs with immune checkpoint inhibitors (ICIs) is also an area of interest. Molecular targeting agents that are beneficial for the survival of patients with HCC often share the same characteristics of anti-angiogenesis, which highlights the importance of this feature in cancer therapy. The restoration of tumor vascular function may help enhance the ability of other drugs that can be used in combination with anti-angiogenic molecules, such as ICIs, to kill tumor cells.

## Advanced progress in immunotherapy in HCC

**Figure 2:**
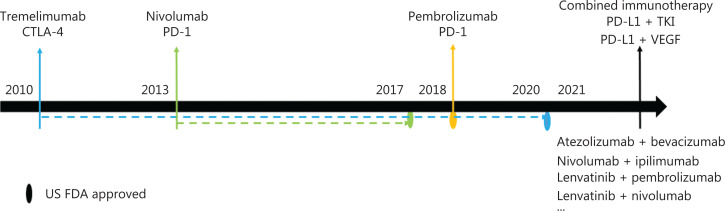
Development of HCC immunotherapy. Ellipses indicate approval by the US FDA.

Immunotherapy has been shown to be effective and safe for the treatment of many solid tumors. The goals of tumor immunotherapy are to control and eliminate tumors by restarting and maintaining the intrinsic immune response, and restoring the body’s normal anti-tumor immune response. Immunotherapies for HCC include: (1) cytotoxic T lymphocyte associated antigen 4 (CTLA-4), (2) PD-1 and its ligand PD-L1, which prevent T cells from recognizing and scavenging cancer cells^137^, and (3) combined immunotherapy (**Figures 1 and 2**).

CTLA-4 plays an important role in the early antigen recognition process and T cell initiation in lymphoid organs. Ipilimumab, an anti-CTLA-4 antibody, was the first approved anticancer drug in this category^138^, whereas tremelimumab, an anti-CTLA-4 antibody, was the first ICI treatment for HCC^139^. Tremelimumab received a rare drug qualification for the treatment of HCC by the U. S. Food & Drug Administration (US FDA), although the development of CTLA-4 blockers for HCC led to the combined application of PD-1 and PD-L1 inhibitors.

PD-1 binds the PD-L1 ligand and prevents T cell activity in peripheral tissues. The overexpression of PD-L1 has been detected in the solid tumor microenvironment, including in HCC. Checkpoint inhibitors are antibodies that activate T cell-mediated antitumor responses by selectively blocking the checkpoint receptors PD-1 and PD-L1^140^.

Nivolumab was the first ICI approved as a second-line treatment for patients with advanced HCC due to disease progression after sorafenib treatment^141^. In a study of genetic markers, PD-1/PD-L1 positivity has been associated with improved prognosis, and an AFP less than 400 μg/L has been associated with potential treatment benefits^78^. Teng et al.^142^ have defined a novel 50-10 rule for AFP response consisting of: CTLA-4 1) a rapid decrease in the AFP response of ≥ 50% of baseline for week 4 (class I) treatment, CTLA-4 2) an AFP change within ± 50% of baseline for week 4 treatment that later declined to ≥ 10% of baseline (class II) or did not (class III) for week 12 treatment, and CTLA-4 3) a rapid increase in AFP of ≥ 50% of baseline for week 4 (class IV) treatment. These criteria have been used to predict the prognosis of patients who received nivolumab monotherapy and those with an AFP delayed response. A rapid decline in the AFP level of more than 50% from the baseline for week 4 treatment has been found to be a predictor of good prognosis^142^. Among 4 patients treated with nivolumab therapy, 2 patients with a clinical response to nivolumab displayed significant decreases in fold changes for their serum *ADAM9* mRNA level. Using The Cancer Genome Atlas database, Oh et al.^143^ have indicated that higher *ADAM9* expression is a potential biomarker for the prognostication of patients with HCC receiving nivolumab. In 45 patients with advanced HCC who received nivolumab after failed treatment with sorafenib, Hung et al.^144^ have explored a biomarker indicating the serum neutrophil-to-lymphocyte ratio and a patient-generated subjective global assessment (PG-SGA) score for guiding nivolumab treatment in patients with HCC. Although many studies have focused on the effects of immunotherapy with nivolumab, biomarkers for predicting tumor responses in patients with HCC remain lacking. In recent years, nivolumab has been used as a first-line treatment for HCC. Although the results have been negative, the OS rate and the response rate for patients with advanced HCC treated with nivolumab have been found to be improved^145^.

Pembrolizumab is another anti-PD-1 antibody approved by the US FDA. Although pebrolizumab treated patients did not reach the preset endpoints for OS and PFS in the phase III confirmatory KEYNOTE-240 trial^146^, this treatment has displayed better ORR and effectiveness in relevant clinical studies^147^. However, the adverse effects and cost efficiency of pebrolizumab are challenging aspects of treatment^80,148^. Camrelizumab is an anti-PD-1 inhibitor developed in China. A phase II clinical study (NCT02989922) has indicated that camrelizumab treatment has antitumor activity in Chinese patients with advanced liver cancer, thereby providing evidence of the effectiveness of PD-1 in the treatment of HBV related liver cancer.

Neoantigens have high cancer specificity and are promising targets for cancer immunotherapy. Continual NGS technology development is enabling development of a comprehensive tumor genome map with the potential to greatly promote the application of new, personalized neoantigens; accurate predictions for new neoantigens are also accelerating the development of personalized immunotherapy^149^.

The combination of immunotherapy and other targeted drugs can effectively improve therapeutic effects^150^. For example, one patient with advanced HCC who received atezolizumab combined with bevacizumab was able to undergo hepatectomy and achieved long-term remission^151^. Recently, the US FDA has approved combination treatment with atezolizumab plus bevacizumab as a breakthrough treatment for untreated advanced or metastatic HCC^152^. Studies have indicated that the ORR for combined immunotherapy is significantly higher than that for the single treatment with any ICI^153–156^. The US FDA has also approved the dual ICI treatment with nivolumab combined with ipilimumab, mainly for patients with HCC previously treated with sorafenib. Radiotherapy and antiangiogenic drugs can influence antigen release or regulate the tumor microenvironment, thereby possibly improving the efficacy of immunotherapy.

The combination of targeted therapy and immunotherapy may lead to good antitumor effects in patients with advanced HCCs^157^. Studies have shown that anti-PD-1 combined with lenvatinib can modulate tumor immunity and enhance antitumor activity within the tumor microenvironment^158,159^. In July 2019, the US FDA approved combined treatment with lenvatinib and pembrolizumab for patients with HCC^160^. Reports of emerging cases have also supported the use of TKI and anti-PD-L1 agents for advanced HCC^161,162^. Kudo et al.^163^ have performed a clinical trial (NCT03418922) and found better effects for lenvatinib plus nivolumab treatment than for lenvatinib plus pembrolizumab treatment in patients with HCC. Consequently, combinations of targeted TKIs plus ICIs may be beneficial for guiding treatment. The exploration of biomarkers for different combined therapies and their prognosis will further promote precision medical treatments for HCC.

Immunotherapy is often accompanied by inevitable adverse effects derived from ICIs, some of which may be very serious^164^. Activation of the immune system can lead to damage in normal healthy tissues, thus leading to a variety of adverse effects including colitis, hepatitis, pneumonia, dermatitis, myocarditis, endocrine gland inflammation, and rheumatic and musculoskeletal phenotypes, such as inflammatory arthritis, arthralgia, myositis, and dryness syndrome^165^. Although some adverse effects during immunotherapy can be alleviated by ceasing use and prescribing steroids, additional immunosuppressants may be required^166,167^. At present, no evidence has indicated that immunosuppressant adverse-effect inhibition affects the antitumor response to ICI treatment.

Strategies for enhancing therapeutic effects and monitoring immunotherapies have been developed with advanced technologies. The advent of immunotherapy and the identification of specific histological and molecular predictors of response to ICIs represent current and future HCC challenges. Only a proportion of patients with HCC appear to benefit from immunotherapy, thus highlighting the need for a deeper understanding of response predictors. In the near future, the HCC medical community should perform additional studies aimed at evaluating novel biomarkers of response to ICIs by considering tumor-intrinsic, immune-specific, and combinatorial biomarkers. In fact, the combination of immunotherapy with other biomarkers would be likely to have a greater influence on HCC treatments than single predictors of response.

## Conclusions and perspectives

With the development of high-throughput sequencing technology and molecular biology, the genomic characteristics of tumors continue to be elucidated. The genomic mutational landscape of HCC and mutational characteristics during disease development have been well documented. However, comprehending the heterogeneity of HCC poses difficulties in clinical treatment. The continual development of new treatment strategies such as targeted therapy, immunotherapy, and various combined therapies within the HCC field; the subtype classification of liver cancer according to molecular characteristics; and the development of biomarkers as guides for choosing therapeutic drugs and determining prognoses may potentially benefit patients with HCC by enabling early diagnosis, accurate treatments, and prognosis monitoring. In the future, these strategies are desirable to support accurate diagnoses and HCC treatments.
